# Gene silencing through RNAi and antisense Vivo-Morpholino increases the efficacy of pyrethroids on larvae of *Anopheles stephensi*

**DOI:** 10.1186/s12936-019-2925-5

**Published:** 2019-08-28

**Authors:** Agata Negri, Marco Ferrari, Riccardo Nodari, Edoardo Coppa, Valentina Mastrantonio, Sergio Zanzani, Daniele Porretta, Claudio Bandi, Sandra Urbanelli, Sara Epis

**Affiliations:** 1 Department of Environmental Biology, Sapienza University of Rome, Via dei Sardi 70, 00185 Rome, Italy; 2 Department of Biosciences and Pediatric Clinical Research Center “Romeo ed Enrica Invernizzi”, University of Milan, Via Celoria 26, 20133 Milan, Italy; 3 Texas Biomedical Research Institute, San Antonio, 7620 NW Loop 410, San Antonio, TX 78227-5301 USA; 4 Department of Veterinary Medicine-DIMEVET, Università degli Studi di Milano, Via Celoria, 10, 20133 Milan, Italy; 5 Centro Interuniversitario di Ricerca sulla Malaria/Italian Malaria Network, Via del Giochetto, 06126 Perugia, Italy

**Keywords:** Insecticide detoxification, ABC-transporter inhibition, Mosquito control, siRNA, Vivo-MO

## Abstract

**Background:**

Insecticides are still at the core of insect pest and vector control programmes. Several lines of evidence indicate that ABC transporters are involved in detoxification processes against insecticides, including permethrin and other pyrethroids. In particular, the ABCG4 gene, a member of the G subfamily, has consistently been shown to be up-regulated in response to insecticide treatments in the mosquito malaria vector *Anopheles stephensi* (both adults and larvae).

**Methods:**

To verify the actual involvement of this transmembrane protein in the detoxification process of permethrin, bioassays on larvae of *An. stephensi,* combining the insecticide with a siRNA, specifically designed for the inhibition of ABCG4 gene expression were performed. Administration to larvae of the same siRNA, labeled with a fluorescent molecule, was effected to investigate the systemic distribution of the inhibitory RNA into the larval bodies. Based on siRNA results, similar experiments using antisense Vivo-Morpholinos (Vivo-MOs) were effected. These molecules, compared to siRNA, are expected to guarantee a higher stability in environmental conditions and in the insect gut, and present thus a higher potential for future in-field applications.

**Results:**

Bioassays using two different concentrations of siRNA, associated with permethrin, led to an increase of larval mortality, compared with results with permethrin alone. These outcomes confirm that ABCG4 transporter plays a role in the detoxification process against the selected insecticide. Moreover, after fluorescent labelling, it was shown the systemic dissemination of siRNA in different body districts of *An. stephensi* larvae, which suggest a potential systemic effect of the molecule. At the same time, results of Vivo-MO experiments were congruent with those obtained using siRNA, thus confirming the potential of ABCG4 inhibition as a strategy to increase permethrin susceptibility in mosquitoes. For the first time, Vivo-MOs were administered in water to larvae, with evidence for a biological effect.

**Conclusions:**

Targeting ABCG4 gene for silencing through both techniques resulted in an increased pyrethroid efficacy. These results open the way toward the possibility to exploit ABCG4 inhibition in the context of integrated programmes for the control *An. stephensi* mosquitoes and malaria transmission.

## Background

Vector-borne diseases are among the main public health threats in the world. According to the WHO, 216 million cases of malaria and 445,000 deaths occurred in 2016 [1]. Although great results in malaria control have been achieved in the past decades, the occurrence of drug resistance in *Plasmodium falciparum*, in particular against artemisinin and other drugs [2–7], and insecticide resistance in mosquito populations [1, 8–11] are threatening the efforts for an effective control of the disease. Insecticides remain the core of all malaria control programmes, despite the diffusion of resistant vector mosquitoes, caused by their heavy use. Pyrethroids, and permethrin in particular, are widely used for indoor residual spraying (IRS) and for the treatment of bed nets. As such, it is of pivotal importance to understand the molecular mechanisms of detoxification in mosquitoes, both in sensitive and resistant populations/strains. This knowledge could lead to the development of strategies aimed to restore sensitivity in resistant populations and to avoid the evolution of resistances in sensitive ones [12–17].

Several studies, carried out over the years, have identified a number of genes involved in the detoxification of xenobiotics in mosquitoes, such as Glutathione-S-transferase (GSTs) [18–22], Epsilon glutathione transferase (GSTe) [23], Cytochromes P450 (CYPs) [22], Acetylcholinesterase (AChE1) [24] and ATP Binding Cassette (ABC) transporters [17, 25–29]. Among these, in particular, the ABCG4 transporter, belonging to the G subfamily of ABCs, has consistently been shown to be up-regulated in response to permethrin treatment, suggesting an important role in detoxification against this insecticide in *Anopheles stephensi* larvae [22, 25, 26] and adults [28]. A first aim of this study was thus to verify whether the ABCG4 efflux pump plays a role in permethrin detoxification in *An. stephensi*. To this purpose, assays using siRNAs targeted on the ABCG4 mRNA, were performed to determine whether the inhibition of the expression of this gene increases susceptibility to permethrin.

It was emphasized that down-regulation through RNA interference (RNAi) has been achieved for various detoxification genes, inducing an increase of mosquito sensitivity to different classes of insecticides [14, 30, 31], but this technique has also been used for identifying new resistance candidate genes [32]. RNAi-based tools have also been tested for their biopesticide potential [33] and as sterility inducer [34]. These results highlight the potential of RNAi as a promising research tool towards the development of novel strategies in vector control. Thus, RNAi-based tools hold potential for possible field applications as larvicides [35].

As stated by the WHO, the larval source management (LSM) still represents the backbone of integrated mosquito control programmes, with a large-scale effectiveness, able to complement measures against adult mosquitoes and limit the residual transmission of malaria [36, 37]. In this backdrop, oligonucleotides for gene expression inhibition, like siRNA for RNAi, gained importance as a potential novel class of ecofriendly larvicides, which can target both insecticide-resistant and-sensible vectors [14, 30, 35]. However, the duration of activity of these molecules and their stability (persistence) in the field, such as the external water environment and the inner organism of the target larvae, has still to be checked [38, 39]. The “oral delivery” of dsRNA to *Anopheles* mosquito larvae, while demonstrating also a systemic knockdown effect of target genes [30], implies a partial degradation of the RNA oligonucleotides in the insect gut [40] and a decrease in their effect [41].

Another antisense gene knockdown technology is the antisense Morpholino (MO), which is based on the action of uncharged molecules able to induce a complementary-based block mRNA translation into protein without degradation of mRNA [39, 42]. The use of these oligonucleotides has achieved excellent results in applications requiring an extreme specificity in complex systems (e.g. embryo development) [39]. These highly stable synthetic oligonucleotides can be also conjugated with a delivery moiety, allowing cell-penetration and the in vivo-uptake. These conjugated molecules, Vivo-Morpholinos (Vivo-MOs), have already been used in cell culture treatment, or in studies in vivo through microinjection [43, 44], electroporation and also through oral administration [45], and bath-immersion [46, 47]. A recent study performed on adult of *An. stephensi* underlined the suitability of Vivo-MO oral delivery as an efficient method for gene knockdown in mosquitoes [41]. For this reason, in the present work the second aim was to confirm the potential of Vivo-MO through administration in water to larvae, verifying the biological effects (larval mortality) and the effects on gene expression.

## Methods

### Mosquito breeding

Eggs derived from a colony of a susceptible strain of mosquitoes, *An. stephensi* Liston strain, are obtained from the insectarium of the University of Camerino, Italy. In this colony, adult and larvae of mosquitoes are reared with a 12:12 light–dark photoperiod, following standard condition of temperature and humidity: 28 ± 1 °C and 85–90% relative humidity, 5% sucrose solution feeding. Eggs are put into well water for hatching and larvae are fed daily with fish food (TetraFish, Melle, Germany), following the same standard conditions of the insectary.

### Specific siRNA design

Two 25nt Stealth RNAi™ siRNA sequences (5′ UCUACACACUGUACUGGCUCAUGUA 3′; 5′ UUUAUCACUCAUCCGAUAUGCCAGG 3′) were designed using the online software BLOCK-IT™ RNAi Designer (Thermo Fisher Scientific, Waltham, Massachusetts, U.S.), with high complementarity to the ABCG4 mRNA available sequence of *An. stephensi* (EMBL accession number: LK392617.1). A scrambled sequence of each siRNA (5′ AUAGCCACAGUGUUAUCUCUUCACG 3′; 5′ GGAAUACGUGUUACCGCAAUUAGAG 3′) without homology to any *An. stephensi* gene has been used as control. Two different siRNAs were administered in order to determine which one was more effective, according to the supplier indications. 5′ UCUACACACUGUACUGGCUCAUGUA 3′ (and relative scramble) was identified as the most effective (data not shown), thus used for further experiments.

### ABCG4 gene silencing in larvae of mosquitoes using siRNA

Treatments with siRNA through oral delivery were performed on the third instar larvae. Groups of 50 third instar larvae were soaked in a volume of 357 μl siRNA, or scrambled siRNA, at two different concentrations (0.03 μg/μl and 0.06 μg/μl) in RNase-free water, to prevent siRNA degradation. The lowest concentration was selected because previously used by Figueira-Mansur et al. [14] on *Aedes aegypti* larvae. Additional groups of 50 larvae were treated only with RNase-free water as a control. This step was performed for 3 h, and fish food (TetraFish, Melle, Germany) was administered to all groups. The 3-h exposure time was determined in a preliminary experiment, soaking *An. stephensi* third instar larvae in 0.5% bromophenol blue according to the protocol described in [31]. At the end of the treatment, each group of larvae was gently transferred in 100 ml of well water and an LD_50_ dose of permethrin (0.072 mg/l) has been added to all groups, except the two control groups [48]. Before the administration, the powdered insecticide was dissolved in acetone and then diluted in water to obtain the test solutions; the LD_50_ and the sub-lethal dose of permethrin (i.e. the dose at which no dead larvae were observed) were determined using different concentrations, as reported in Epis et al. [25]. All the experiments were performed three times.

### Gene expression analysis in larvae treated with siRNA

After six and 24 h of permethrin exposure, pools of 5 surviving individuals (able to move through the water column) were put in extraction buffer + β-mercaptoethanol for immediate RNA extraction using the commercial RNeasy Mini Kit (Qiagen, Hilden, Germany) with an additional on-column DNase I treatment (Qiagen, Hilden, Germany), according to the manufacturer’s instructions. RNA concentration was determined by Qubit 3.0 Fluorometer (Thermo Fisher Scientific, Waltham, Massachusetts, U.S.). cDNAs were synthesized starting from 200 ng of total RNA, using a QuantiTect Reverse Transcription Kit (Qiagen, Hilden, Germany) with random hexamers. cDNA was used as template in RT-qPCR reaction, using ABCG4 primers, already published in previous works [25, 26]. Two endogenous reference genes for *An. sthephensi* were used to obtain a normalization of data: *rps7* [49] and GAPDH [50] (Table 1). Gene relative expression was determined using a BioRad iQ5 Real-Time PCR Detection System (Bio-Rad, California, USA). The analysis was carried out in accordance with the following conditions: 50 ng cDNA; 300 nM of forward and reverse primers; 98 °C for 30″, 40 cycles of 98 °C for 15″, 59 °C for 30″, 72 °C for 30″; fluorescence acquisition at the end of each cycle; melting curve analysis after the last cycle. Cq values were determined for each gene, in order to calculate gene expression levels of target gene using *rps7* and GAPDH as internal reference genes. The expression level of ABCG4 in the control group was considered as basal level, in order to evaluate the effect of permethrin induction and RNAi effect. The estimates of the expression level of ABCG4 in the siRNA- treated and scramble-treated larvae are reported as means between different pools ± standard deviation (SD).

**Table 1:** Primer sequences of ABC transporters and housekeeping genes of *Anopheles stephensi*

Gene	Forward primer	Reverse primer	bp	Sources
*Anst*ABCG4	ATGAGCCCATTCGTCCTG	AGCGTGGAGAAGAAGCAG	158	[25]
*rps7*	AGCAGCAGCAGCACTTGATTTG	TAAACGGCTTTCTGCGTCACCC	90	[49]
GAPDH	GCCGTCGGCAAGGTCATCCC	TTCATCGGTCCGTTGGCGGC	166	[50]

### Mortality bioassay on larvae treated with ABCG4 siRNA

In order to estimate the mortality of larvae, induced by the combined treatment of permethrin and siRNA, a specific bioassay was performed. Briefly, groups of 25 larvae were soaked in 178 μl of siRNA or scrambled siRNA at a concentration of 0.03 μg/μl and 0.06 μg/μl in RNase free water. Groups of larvae were treated with siRNA or scramble siRNA, while additional groups were treated only with water. After 3 h of treatment each group was gently transferred into 100 ml of well water plus an LD_50_ of permethrin (0.072 mg/l), previously determinate with other bioassays with different insecticide concentration. Groups of 25 larvae soaked in just water were used as control. Mortality was assessed after 6 h and 24 h of permethrin exposure and larvae were considered dead if static, even after a mechanical stimulus [48].

### Systemic dissemination of siRNA

To verify if the ABCG4 siRNA was able to be absorbed into the larva (in particular into the midgut) third instar larvae were soaked for 3 h in the higher concentration (0.06 μg/μl) of the same ABCG4 siRNA, conjugated with Alexa Fluor 488 (Thermo Fisher Scientific, Waltham, Massachusetts, U.S.). The diffusion of the fluorescent signal was analysed by Zeiss Axio Zoom.V16 stereo microscope after 3 h and 24 h of siRNA exposure.

### Specific Vivo-Morpholino design

An ABCG4 Vivo-Morpholino (AnstMO_ABCG4; GeneTools LLC, Philomath, OR, USA) was designed using the Tool of GeneTools LLC company and it was comprised of a Morpholino conjugated to a transporter structure/delivery moiety, comprised of an octa-guanidine dendrimer, that improves uptake of the oligonucleotides by cells in tissues [51]. The sequence (5′ ATGCTCTAGCTTCTCGCACACCAAA 3′) of the Vivo-Morpholino AnstMO_ABCG4 was designed with high complementarity to the ABCG4 mRNA sequence of *An. stephensi* (accession number LK392617.1) following the suggestion of the manufacturers. As for the siRNA, the Vivo-Morpholino AnstMO_ABCG4 was administered at the third instar larvae [48] in nuclease free water.

### ABCG4 gene silencing in larvae of mosquitoes using Morpholino

This is the first study where the Vivo-Morpholino oligonucleotides has been used against mosquito larvae; for this reason, the experimental procedure for oral administration through bath immersion were performed following the protocol previously reported for siRNA experiments. About the concentrations, different doses of AnstMO_ABCG4 (0.051 μg/μl; 0.101 μg/μl; 0.203 μg/μl; 0.406 μg/μl) were tested, in order to evaluate the necessary concentration to obtain the downregulation effect in larvae, through oral feeding. This pre-test (results not shown) led to the definition of two efficient concentrations, 0.203 μg/μl; 0.406 μg/μl. Following the previous protocol, groups of 50 third instar larvae were soaked for 3 h in a volume of 357 μl of RNase-free water plus AnstMO_ABCG4 at 0.203 μg/μl or 0.406 μg/μl. Control groups were treated only with RNase-free water. Thereafter, each group of larvae was treated, for 6 and 24 h, with the LD_20_ dose (0.030 mg/l) of permethrin in 100 ml of well water. The experiment was performed in three times.

### Gene expression analysis in larvae treated with Morpholino

Pools of five surviving larvae were collected at six and 24 h and soaked in extraction buffer + β-mercaptoethanol for RNA extraction. The analyses of gene expression were carried out following the previously described procedure.

### Mortality bioassay on larvae treated with Morpholino

Mortality bioassay was performed to evaluate the phenotypic effect on larvae treated with the combination of permethrin and Vivo-Morpholino. The same protocol described for the siRNA-bioassays was applied using the AnstMO_ABCG4 at two concentrations, 0.203 μg/μl and 0.406 μg/μl.

### Statistical analysis

In the bioassays (after six and 24 h) effects of previously described treatments on mosquitoes’ mortality and ABCG4 gene expression levels were compared by a one-way ANOVA; when analysis of variance resulted statistically significant (p < 0.05), post hoc comparisons were performed by Least Significance Difference (LSD). All analyses were implemented using the SPSS software (version 20.0; SPSS, Chicago, Illinois).

## Results

### Bioassays on larvae after siRNA treatment

**Fig. 1:**
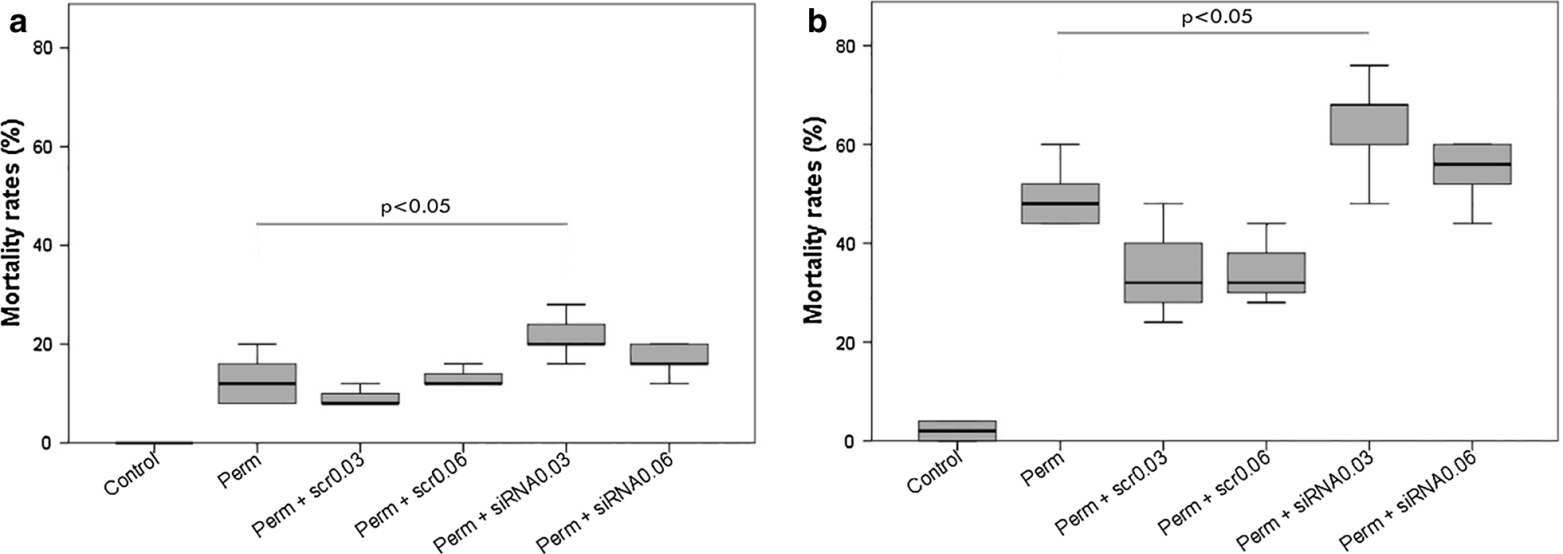
Larval mortality rates in siRNA bioassays. Mortality obtained after 6 h (**a**) and 24 h (**b**) of LD_50_ permethrin exposure in silenced and non-silenced larvae of *An. stephensi*, through two different concentrations of siRNA (0.03 μg/μl and 0.06 μg/μl). Data were compiled from three time replicate experiments and assessed by one-way ANOVA with *Post*-*Hoc* LSD test as multiple comparison test. p < 0.05 in comparison to permethrin treated larvae; error bars denote standard deviation of the means (SD)

After determination of the LD_50_ of permethrin (0.072 mg/l at 24 h), the mortality of larvae was assessed at six and 24 h. Using the LD_50_ concentration, permethrin treatment induced a 12.8 ± 5.21% (mean ± SD) mortality after 6 h; when administered following the 0.03- or 0.06-μg/μl siRNA treatments, a 21.6 ± 4.6% or 20 ± 9.38% mortality was observed, respectively. A similar pattern was observed after 24 h, where permethrin alone led to a 45.6 ± 13.45% mortality, increased to 64 ± 10.58% and 58.4 ± 13.45% by siRNA pre-treatments (at the 0.03- and 0.06- μg/μl concentrations). The differences between the treatment with permethrin alone and those added with the lowest concentration of siRNA were statistically significant at both time points (LSD test p = 0.027 after 6 h p = 0.024 after 24 h). Moreover, at 24 h the difference in the efficacy of the scrambled siRNA compared with the gene specific siRNA was also statistical significance (LSD test p = 0.003 in comparison to 0.03 μg/μl siRNA; p = 0.013 in comparison to 0.06 μg/μl and siRNA) even, at both concentrations (Fig. 1).

No statistical differences were observed between the permethrin alone treatment and those with the two doses of scrambled siRNAs (0.03 μg/μl and 0.06 μg/μl) (respectively p = 0.422 and p = 0.901 after 6 h; both p = 0.219 after 24 h) (Fig. 1).

### ABCG4 relative expression in larvae after siRNA treatment

**Fig. 2:**
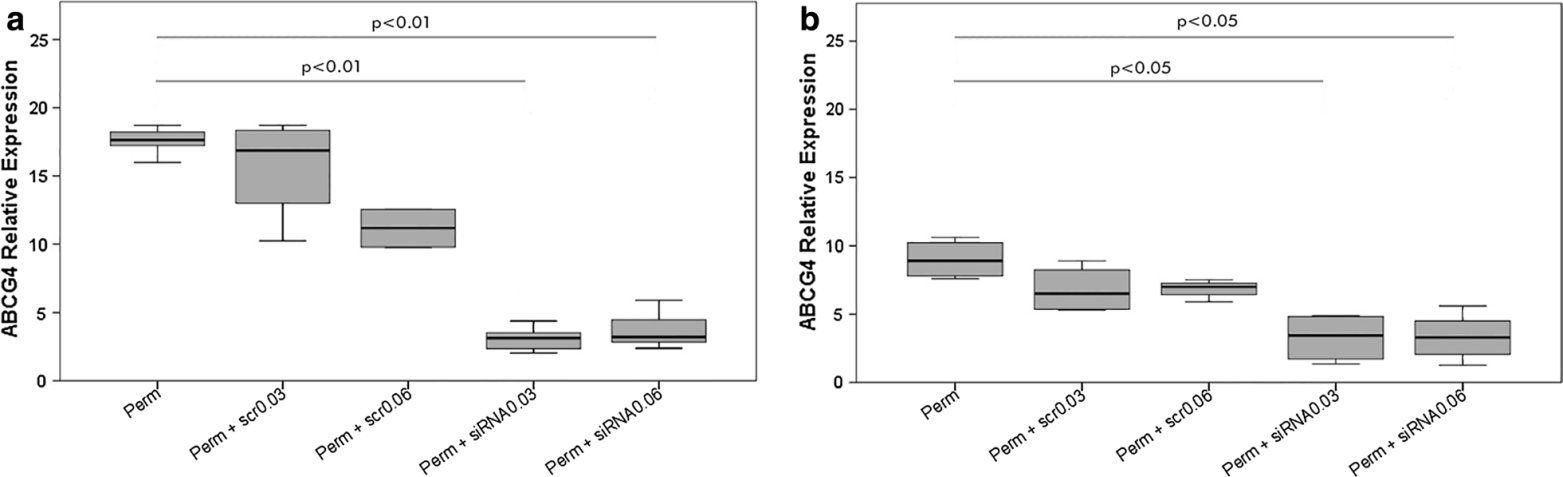
ABCG4 relative expression after siRNA and permethrin treatments. ABCG4 induction after 6 h (**a**) and 24 h (**b**) of LD_50_ permethrin exposure in silenced and non-silenced larvae of *An. stephensi,* through two different concentrations of siRNA (0.03 μg/μl and 0.06 μg/μl). Data were compiled from three time replicate experiments and assessed by one-way ANOVA with *Post*-*Hoc* LSD test, as multiple comparison test. p < 0.01 in comparison to permethrin treated larvae (**a**); p < 0.05 in comparison to permethrin treated larvae (**b**); error bars denote standard deviation of the means (SD)

The relative expression of ABC4 transporter was assessed through qRT-PCR on larvae exposed to the LD_50_ of permethrin, alone or in combination with siRNAs and scrambled siRNAs, After 6 h, permethrin induced an up-regulation of ABCG4 of 17.56 ± 1.04-fold, while pretreatment with siRNA, at the two concentrations, led to an up-regulation of only 3.08 ± 0.94 and 3.76 ± 1.34 respectively, when compared with control. In other words, a “downregulation”, compared to the insecticide alone, of 15- or 13-folds, respectively, for the 0.03 μg/μl and for 0.06 μg/μl siRNA pre-treatments, have been observed. Post hoc LSD test showed that permethrin treated larvae did not significantly differ in ABCG4 expression compared with those treated with scramble siRNA (15.67 ± 3.83 and 11.17 ± 1.94 fold), while a significant difference was observed with the two siRNA concentrations (p = 0.005 for the higher and p = 0.008 for the lower concentration). Whereas the two concentrations of siRNA did not show a statistically significant difference between them (Fig. 2a).

After 24 h, the insecticide induced an up-regulation of 9.01 ± 1.43-fold in permethrin-treated larvae compared to the controls. Permethrin in combination with the two siRNA concentrations led to a reduced up-regulation, of 3.27 ± 1.83 and 3.34 ± 1.76-fold respectively for the lower and higher concentrations, compared to control. In other words, pre-treatments with siRNA in addition to the insecticide showed a down-regulation of 5.74- and 5.67- fold for the lower and higher concentrations, when compared with insecticide-alone treatment (Fig. 2b). The post hoc LSD test highlighted no significant differences between ABCG4 expression in permethrin-treated larvae compared to those treated with scramble siRNA (which showed a 6.81 ± 1.75 and 6.86 ± 0.68-fold expressions relative to control). On the other hand, a significant difference was detectable with the two siRNA concentrations, also at the 24 h time point (p = 0.028 and p = 0.035 respectively for the lower and higher concentrations). No statistically significant difference was detected between the two concentrations of siRNA.

### Systemic siRNA diffusion in *Anopheles stephensi* larvae

**Fig. 3:**
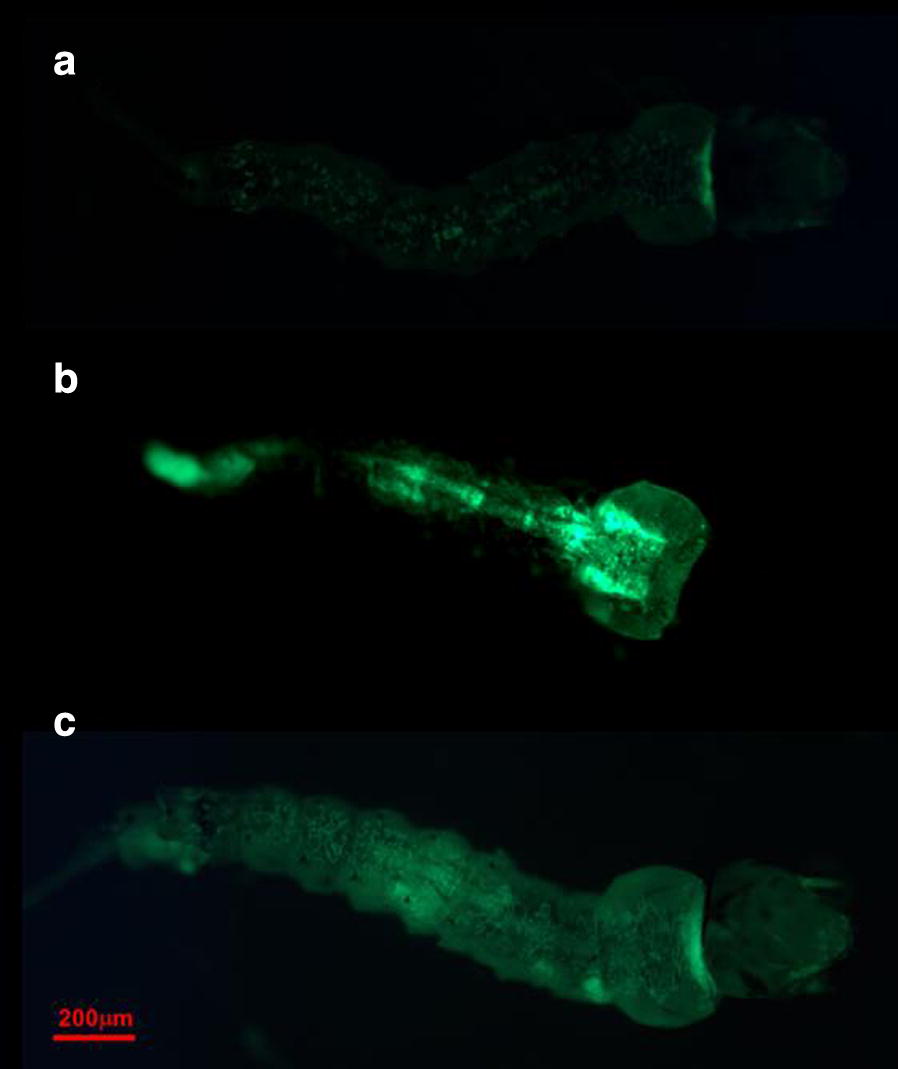
Fluorescence emission in siRNA (Alexa Fluor 488) bioassays. Third instar larvae of *An. stephensi* exposed to a 0.06 μg/μl concentration of a fluorescent siRNA. **a** Control larva with slight autofluorescence. **b** Larva after 3 h of exposure to the siRNA; the fluorescence is concentrated at the gut level. **c** Larva exposed for 3 h to the siRNA and transferred for 24 h in water. The signal is diffused to the whole body

Third instar larvae were exposed to the 0.06 μg/μl concentration of the fluorescent siRNA (Alexa fluor 488) for 3 h to allow them to up-take the molecule. Figure 3 shows that, after 3 h of exposure, fluorescence is localized mainly in the gut, in the central part of the larval body (Fig. 3b), coherently with the assumption that siRNA molecule is acquired by the larvae through the oral route. The fluorescence emission is much more evident in the treated larvae compared to the controls (Fig. 3a), in which the emission can be interpreted as auto-fluorescence. After the 3 h of exposure, larvae were moved into fresh water for 24 h and then analysed. In this case the fluorescent signal is detectable in all the tissues of the specimens (Fig. 3c).

### Bioassays on larvae after Vivo-MO treatment

**Fig. 4:**
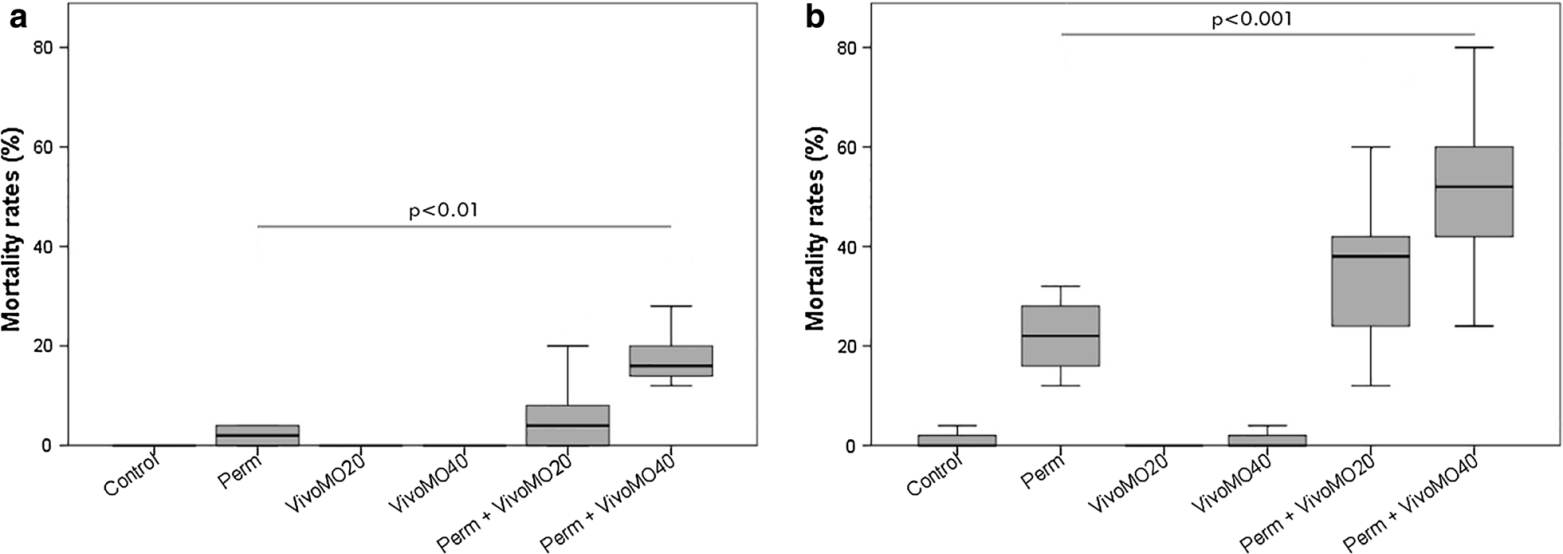
Larval mortality (%) in Vivo-MO bioassays. After 6 h (**a**) and 24 h (**b**) of LD_20_ permethrin exposure in silenced and non-silenced larvae of *An. stephensi,* through two different concentrations of Vivo-MO (20 μM = 0.203 μg/μl and 40 μM = 0.406 μg/μl). Data were compiled from three time replicate experiments and assessed by one-way ANOVA with *Post*-*Hoc* LSD test, as multiple comparison test; p < 0.01 and p < 0.001 in comparison to permethrin treated larvae; error bars denote standard deviation of the means (SD)

Figure 4 shows the results of the in Vivo-MO bioassays, that were effected with a sub-lethal dose of permethrin (LD_20_). In groups of larvae treated with Vivo-MO alone, the oligonucleotides, at the two concentrations, did not affect the larval survival: at both time points and at both concentrations mortality was equal to that of the controls (0% at 6 h and 1.33 ± 2.3% at 24 h). In contrast, treatment with Vivo-MO combined with permethrin led to an increase in larval susceptibility, with a dose-dependent effect that raise over time. At 6 h (Fig. 4a), larval mortality with permethrin alone was only 2 ± 2.3% (mean ± SD); after exposure to the two concentrations of Vivo-MO, 0.203 μg/μl, and 0.406 μg/μl, permethrin-determined mortality increased up to 6.67 ± 7.88% and 18.33 ± 6.26%. At 24 h (Fig. 4b), a fortiori, the 22 ± 8.33% mortality, determined by the sub-lethal treatment with insecticide alone, increased up to 34.67 ± 13.89% with the lowest dose of Vivo-MO, and reached the 51.67 ± 15.95% with the highest dose. Therefore, at 24 h, an increase in the mortality rate of 13% was achieved with 0.203 μg/μl of Vivo-MO, and an increase of 30% with 0.406 μg/μl of Vivo-MO. The post hoc LSD test showed a statistically significant difference (p = 0.0032 after 6 h; p = 0.0006 after 24 h) between the permethrin treatment alone and the one with the highest dose of Vivo-MO at both time points.

### ABCG4 relative expression analysis in larvae after Vivo-MO treatments

**Fig. 5:**
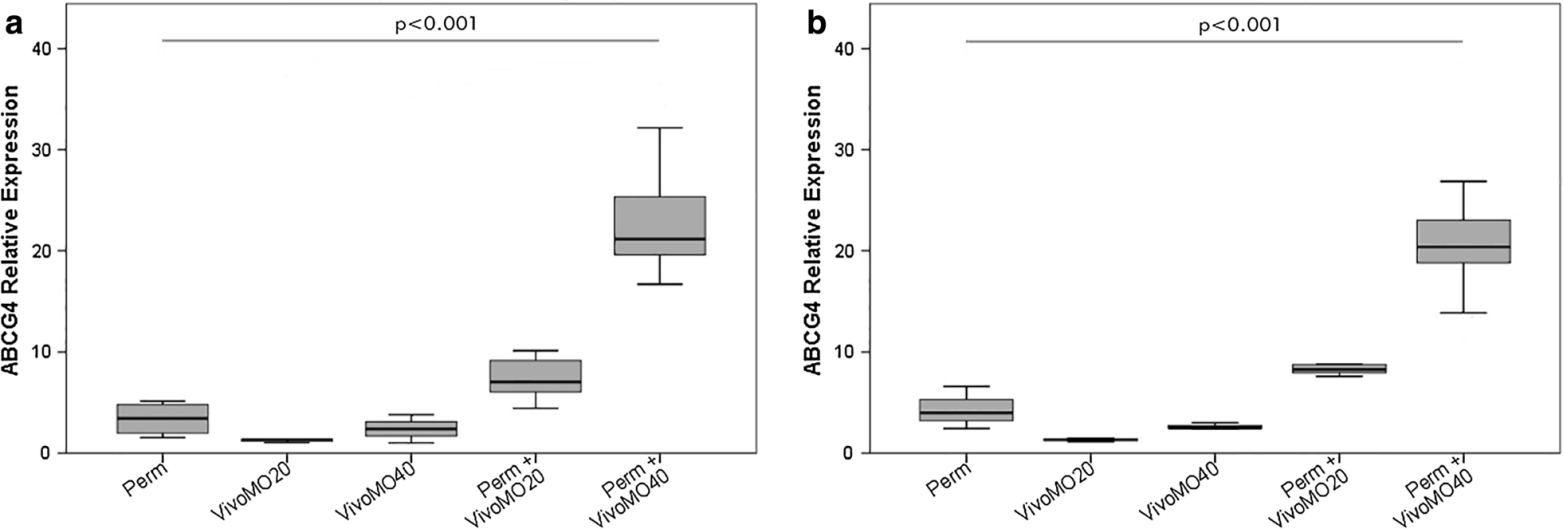
ABCG4 relative expression after Vivo-MO and permethrin treatments. ABCG4 induction after 6 h (**a**) and 24 h (**b**) of LD_20_ permethrin exposure in silenced and non-silenced larvae of *An. stephensi,* through two different concentrations of Vivo-MO (20 μM = 0.203 μg/μl and 40 μM = 0.406 μg/μl). Data were compiled from three time replicate experiments and assessed by one-way ANOVA with *Post*-*Hoc* LSD test, as multiple comparison test. p < 0.001 in comparison to permethrin treated larvae; error bars denote standard deviation of the means (SD)

Gene expression analysis was performed through qRT-PCR on groups of larvae, after six and 24 h of treatment with or without Vivo-MO, followed by the treatment with LD_20_ dose of permethrin (except for Vivo-MO alone treatment) (Fig. 5). Considering the mechanism of action of Morpholinos, the molecule binds/inhibits a specific target mRNA sequence, without degrading it, and so it could even induce an ABCG4 upregulation, being a xenobiotic compound, instead of a downregulation. At 6 h the relative expression of ABCG4, compared to the control, showed a lower value for larvae treated with permethrin alone, of 3.38 ± 1.7, than larvae treated with the combination of insecticide plus Vivo-MO at 0.203 μg/μl and 0.406 μg/μl, with values of 7.38 ± 2.04 and 23.97 ± 6.81 respectively. The target gene after inhibition with MO oligonucleotides, in fact, was up-regulated 4.00 fold more than permethrin alone, by the lowest concentration of oligonucleotides, and 20.59 fold by the highest concentration. On the contrary, the ABCG4 expression in the treatments with Vivo-MO alone (of 1.25 ± 0.17 fold with 0.203 μg/μl and 2.40 ± 1.40 fold with 0.406 μg/μl) is lower than those of treatment with inhibitor plus insecticide, and that of treatment with permethrin alone. Using the post hoc LSD test it was possible to detect the differences in relative expression for all treatments. A statistically significant difference was observed between the expression induced by 0.406 μg/μl Vivo-MO (p = 0.0006) and that of all other treatments, including the permethrin-alone treatment and the 0.203 μg/μl Vivo-MO treatment.

The expression levels didn’t face evident changes from six to 24 h: at 24 h the higher dose of Vivo-MO caused a slight decrease of ABCG4 expression (not statistically significant), perhaps due to the weakening of cellular defenses over time. Relative expression of permethrin was of 4.25 ± 1.72 fold, compared to permethrin in combination with Vivo-MO at lowest concentration, (9.07 ± 2.26) and at the highest concentration (19.04 ± 8.43). This highlights an up-regulation of 4.82 and 14.79 respectively, when compared with insecticide alone treatment. The multiple comparisons analysis showed that permethrin treatment did not differ from those with Vivo-MO alone (p = 0.355 with the low concentration; p = 0.609 with the high concentration), while a significant difference was obtained with the combination of insecticide plus the higher concentrations of Vivo-MO [LSD tests (p = 0.0007)].

## Discussion

As for our results about the RNAi, the expression analysis on the whole larval body are consistent with the data from previous studies, showing that the peak of ABCG4 over-expression occurs after 6 h of exposure to permethrin [26]. This transporter was chosen as a target for silencing because it demonstrated a strong up-regulation among the ABC transporters in *An. stephensi* in response to permethrin treatment, suggesting its involvement in the detoxification against this insecticide [22, 25, 26]. RNAi-based assays were thus performed to confirm this hypothesis, thus to assess whether the inhibition of ABCG4 has an effect on mosquito mortality, using two siRNA concentrations. At both concentrations, at 0.03 μg/μl and 0.06 μg/μl, siRNAs were able to induce an increased mortality at the two examined time-points (Fig. 1). This increase is correlated with the RNAi efficacy in down-regulating the expression of ABCG4 at both time points at both concentrations (Fig. 2). In this study, a detectable down-regulation using both the 0.03 μg/μl and the 0.06 μg/μl doses was achieved. In particular, after 24 h a down-regulation of around 5.74-fold, compared with insecticide-alone treatment, was achieved with the lower concentration; this downregulation in gene expression was associated with an almost 20% mortality increase. These obtained results are consistent with studies where RNAi targeted on efflux pumps and G-protein-coupled receptor of insecticide resistant larvae of *Aedes aegypti* and *Culex quinquefasciatus* induced an increased toxicity, respectively, of temephos and permethrin [14, 52, 53]. These results, and those of the current study, are also coherent with another work on the diamondback moth *Plutella xylostella*, where the silencing of another ABC transporter, ABCH1 (structurally similar to ABCG sub-family members), led to larval and pupal lethal phenotypes when exposed to the Cry toxin of *Bacillus turingiensis* [54]. By comparing the results obtained at the two times and at the two concentrations, the increase in larval mortality was not correlated with the dose of siRNA used or with the duration of insecticide exposure (Fig. 2). It is thus possible that at 6 h of exposure, with the 0.03 μg/μl siRNA dosage, a plateau effect is achieved.

With the prospect of applying gene silencing tools in the field, for the control of mosquito larvae, an important issue to be addressed is the method of delivery of interfering/downregulating molecules. The results using siRNA show that the administration in water, and thus the acquisition of the molecule via oral feeding, work successfully on *An. stephensi* larvae, as previously shown for other mosquito species [31] as well as for insect species belonging to different orders [55]. Despite this, the possible factors that could affect the downregulation and, therefore, the achievement of a high level of larval mortality, remain to be clarified. The efficiency of RNAi is highly variable in insect species and in different conditions: the critical factors that determine the ability of the target organism to uptake the oligonucleotides, its spread to tissues and cells, the activation of an autonomous RNAi machinery for mRNA degradation should specifically be investigated in the different species [56]. Moreover, the critical factors related to the molecule, e.g. its stability and integrity in the environment and in the larval gut lumen, needed to guarantee the cellular delivery of a sufficient amount of intact siRNA, have not yet been investigated in detail [40]. For example, about host-related factors, recent studies suggested that Diptera lack the transmembrane channel-mediated uptake mechanism, formed by the RNA channel transporters SID-1 and SID-2, that are involved in siRNA uptake in the gut lumen of other insects [55, 57–60]. Despite this, the results with fluorescent siRNA demonstrate that this type of molecule actually diffuses into the larval mosquito body, possibly through an endocytosis-mediated mechanism, as suggested in other studies on larvae of *Aedes aegyp*ti and *Anopheles gambiae*, that demonstrated the spread of the RNAi effects, after oral administration, to tissues outside the gut [30, 31]. Anyway, the clear downregulation of ABCG4 expression in response to siRNA, observed at both six and 24 h of permethrin treatment (for both concentrations used), indicates that the molecule actually reaches mosquito cells, activating the degradation of target mRNA also in *An. stephensi* larvae.

Evidence for a systemic RNAi in dipterans has already been obtained in other studies which indicated an efficient internalization and biodistribution of dsRNA in *An. gambiae* cultured cells [61], and a strong RNAi effect throughout the development of mosquitoes in *Aedes aegypti* larvae, after feeding [34]. Aspects that are still to be clarified regarding the actual amount of siRNA that reaches larval tissues, after the administration through the breeding water, with the various possible “challenges”, such as pH of the water and gut lumen, and the potentially variable conditions of the gut lumen of larvae in field conditions [40]. The exposure of oligonucleotides to these challenges, as noted in different reports [31, 62], implies that oral feeding-based delivery of interfering RNA molecules determines a less effective knockdown compared to microinjection-based administration. On the other hand, oral administration of siRNA is less time-consuming than microinjection, and more suitable for high-throughput screenings. In addition, it is likely the sole possible way of delivery for field applications for mosquito control.

Recently, to achieve the oral administration of RNAi-inducing molecules to be tested as biopesticides, different approaches have been developed with the aim of delivering intact dsRNAs/siRNAs into cells [63]. The study of abiotic and biotic transport methods was designed to prevent the degradation of RNA molecules, more frequently in the field settings than in lab conditions [64, 65]. Liposomes were tested as abiotic carriers [66, 67], as well as hydrogel- [61], carbon quantum dot- [68] and chitosan-nanoparticles [35, 69]; the last ones, for example, are currently considered as the most economical and environmentally safe system for the delivery of dsRNAs to insect larvae [64, 70]. On the other side, the alternative biotic delivery was achieved through the *Escherichia coli* and the *Pichia pastoris* expression systems, for a cheaper large-scale administration of RNAi inducing molecules to third instar larvae of mosquitoes [71, 72].

Among the antisense gene knockdown technologies, antisense Morpholinos (MOs) present characteristics that should guarantee key advantages for future field applications. They can ensure highly specific antisense activity, and are able to bind the target RNA sequence having less interaction with unintended mRNAs, compared with knocking-down RNAi methods based on protein/catalytic activity (e.g. siRNA). Their stability is due to their molecular structure, and to the covalently linked delivery moiety, that make them more durable in water, and more easily internalized by intestinal cells [42]. Moreover, their lack of electrostatic charge (neutral charge) minimizes the interaction with proteins, hence implying a reduced toxicity and immunogenicity; at the same time, being chemically different from a “normal” DNA or RNA molecules, they are resistant to nucleases, which implies a higher stability [39, 42].

The results here obtained showed an increased susceptibility to permethrin after Vivo-MO treatment, congruent with the results obtained using siRNA; the role of the ABCG4 transporter in permethrin detoxification in *An. stephensi* is thus further supported. The addition of Vivo-MO to the LD_20_ dose of permethrin led to a significant increase of larval susceptibility, until reaching a 50% mortality at the second time point. In particular, after 24 h, the 0.406 μg/μl Vivo-MO dose determined an increase in larval mortality of 30% compared to permethrin alone, while the 0.203 μg/μl dose, at the same time, determined an increase in mortality of 13% (Fig. 4b). The reached effect appears dose- and time-dependent, with mortality of the two increasing doses shifting from 10 to 23% at 6 h and from 35 to 52% at 24 h. As shown by our results, administration of Vivo-MO in absence of permethrin did not cause any detectable/significant effect in term of mortality and ABG4 gene expression. It could be concluded that the molecule is non toxic if administered alone: its effects were detrimental to mosquito larvae when administered with permethrin. This feature has primary importance to avoid negative effects on non-target organisms: specifically-designed Vivo-MO should have no effects on non-target species.

As for the relative expression of ABCG4 gene (Fig. 5a, b), Vivo-MOs act differently from siRNAs, making mRNA unavailable for translation, but without degrading it: mRNA molecules, bounded and inhibited by the oligonucleotides, likely accumulate inside the cell without being used [38]. It was actually observed that ABCG4 mRNA amount increased after combined permethrin-Vivo-MO treatment (Fig. 4a, b), while siRNA-permethrin treatment lead to a reduction in ABG4 mRNA content in the larvae (Fig. 2a, b). It is possible to suggest that, after the permethrin + Vivo-MO treatment, the larval organism is increasingly stimulated to produce ABCG4 transporter to expel the insecticide, but the mRNA overproduced has no effect, as it has been seen from the mortality results (Fig. 3a, b): the more Vivo-MO molecules bind the target mRNA, making it inactive, the greater the production of mRNA. The expression of ABCG4 gene, as can be seen from the comparison between the two time points, with the two concentrations of Vivo-MO, is stable (expression at six and 24 h is not different), but is dose-dependent, i.e. with the higher dose of Vivo-MO the amount of mRNA detected is higher. It is thus possible that the administration of a higher dose of Vivo-MO in the presence of the same dose of permethrin would lead to an increased larval susceptibility to permethrin. However, the possibility that inhibition of ABCG4 could lead to the activation of other defensome genes responsible for detoxification processes and cellular defence (an issue worth of further investigations [17, 22]), should be considered. Due to the lack of *ad*-*hoc* antibody against the ABCG4 of mosquitoes (or other insects), quantification of its expression at the protein level is challenging. Therefore, or goal was to determine the effect of this nucleotide on larval mortality (a sort of phenotypic effect). In future studies, it would also be interesting to design a scramble Vivo-MO control, not complementary to the target gene, in order to assess whether the phenotypic effect is really due to the lack of the transporter or to any other phenomena. For example, in previous studies, it has been shown that permethrin can strongly interact with nucleic acids, intercalating DNA bases (it is prone to bind to G-C base pairs) [73]. One might thus suggest that the effects that were recorded could have partially been determined by this, or similar, effects of Vivo-MOs.

In the wild, mosquito larvae are exposed to the residues of insecticides used in agriculture or in the control of adults, where these residues flow to the breeding sites [7]. These low amounts of insecticides, creating a toxic stress, could induce tolerance by the upregulation of genes involved in the xenobiotic metabolism/detoxification of chemicals [74, 75]. Notably, this low dose insecticide exposition is regarded as one of the major causes for the onset of resistant forms in mosquitoes [76, 77]. Considering the above phenomenon, it was decided to perform Vivo-MO assays using a low permethrin dosage (LD_20_), which might represent a condition present in breeding sites in treated areas.

Since prior to this study no bioassays had been carried out with Vivo-MO on mosquito larvae, the doses and the timing of administration applied in this study represent a first methodological reference for antisense MO application through direct feeding in mosquito larval stages. It will certainly be interesting to verify in the future if a higher dose of the molecule could lead to a further increase in the susceptibility to permethrin. The possibility to prolong the knocking-down with sequential Vivo-MO treatments against the same ABC transporter should be tested, as well as a multiple knockdown targeting different detoxification genes, in order to avoid that the MO effect declines over time, or to block compensatory effects [41].

## Conclusion

In this study, the effects of two different types of mRNA-targeting oligonucleotides, designed to inhibit the expression of the defensome gene ABCG4, and administered through oral delivery in water, were determined on larvae of *An. stephensi*. The obtained results shown that, targeting ABCG4 gene for silencing through both techniques resulted in an increased pyrethroid efficacy. In conclusion, these results open the way toward the possibility to exploit the inhibition of this gene in the context of integrated programmes for the control of *An. stephensi* mosquitoes and thus malaria transmission. Of course, prior to field application, several issues should be addressed. First, the potential effects on non-target organisms, starting with in silico studies on the specificity of siRNA or Vivo-MO oligonucleotides, but also addressing their potential “side effects” not related with mRNA targeting. Secondly, the stability and methods of delivery of these molecules in field conditions. Finally, field application would obviously require a cost-effective method for production of the oligonucleotides.

## Data Availability

The datasets used and/or analysed during the current study are available from the corresponding author on reasonable request.

## Abbreviations

siRNA: short interference RNA
Vivo-MO: antisense Vivo-Morpholino
RNAi: RNA interference

## References

[1] WHO. World malaria report 2018. Geneva: World Health Organization; 2018. http://www.who.int/iris/handle/10665/275867.

[2] Phyo AP, Ashley EA, Anderson TJC, Bozdech Z, Carrara VI, Sriprawat K (2016). Declining efficacy of artemisinin combination therapy against *P. falciparum* malaria on the Thai–Myanmar border (2003–2013): the role of parasite genetic factors. Clin Infect Dis..

[3] Fairhurst RM, Dondorp AM (2016). Artemisinin-resistant *Plasmodium falciparum* malaria. Microbiol Spectr..

[4] Tilley L, Straimer J, Gnädig NF, Ralph SA, Fidock DA (2016). Artemisinin action and resistance in *Plasmodium falciparum*. Trends Parasitol..

[5] Ouji M, Augereau JM, Paloque L, Benoit-Vical F (2018). *Plasmodium falciparum* resistance to artemisinin-based combination therapies: a sword of Damocles in the path toward malaria elimination. Parasite..

[6] Nair S, Li X, Arya GA, McDew-White M, Ferrari M, Nosten F (2018). Fitness costs and the rapid spread of kelch13-C580Y substitutions conferring artemisinin resistance. Antimicrob Agents Chemother.

[7] WHO. World malaria report 2016. Geneva: World Health Organization; 2016. http://www.who.int/iris/handle/10665/252038.

[8] Karunamoorthi K (2011). Vector control: a cornerstone in the malaria elimination campaign. Clin Microbiol Infect.

[9] Tikar SN, Mendki MJ, Sharma AK, Sukumaran D, Veer V, Prakash S (2011). Resistance status of the malaria vector mosquitoes, *Anopheles stephensi* and *Anopheles subpictus* towards adulticides and larvicides in arid and semi-arid areas of India. J Insect Sci..

[10] Alonso PL, Tanner M (2013). Public health challenges and prospects for malaria control and elimination. Nat Med.

[11] Alout H, Labbé P, Chandre F, Cohuet A (2017). malaria vector control still matters despite insecticide resistance. Trends Parasitol..

[12] Buss DS, Callaghan A (2008). Interaction of pesticides with p-glycoprotein and other ABC proteins: a survey of the possible importance to insecticide, herbicide and fungicide resistance. Pestic Biochem Physiol.

[13] Porretta D, Gargani M, Bellini R, Medici A, Punelli F, Urbanelli S (2008). Defence mechanism against insecticides temephos and diflubenzuron in the mosquito *Aedes caspius*: the P-glycoprotein efflux pumps. Med Vet Entomol.

[14] Figueira-Mansur J, Ferreira-Pereira A, Mansur JF, Franco TA, Alvarenga ES, Sorgine MH (2013). Silencing of P-glycoprotein increases mortality in temephos-treated *Aedes aegypti* larvae. Insect Mol Biol.

[15] Lima EP, Goulart MOF, Rolim-Neto ML (2014). Evaluation of the role of ATP-binding cassette transporter as a defence mechanism against temephos in populations of *Aedes aegypti*. Mem Inst Oswaldo Cruz.

[16] Dalla Bona AC, Faitta Chitolina R, Lopes Fermino M, de Castro Poncio L, Weiss A, Pereira Lima JB (2016). Larval application of sodium channel homologous dsRNA restores pyrethroid insecticide susceptibility in a resistant adult mosquito population. Parasit Vectors..

[17] Pignatelli P, Ingham VA, Balabanidou V, Vontas J, Lycett G, Ranson H (2018). The *Anopheles gambiae* ATP-binding cassette transporter family: phylogenetic analysis and tissue localization provide clues on function and role in insecticide resistance. Insect Mol Biol.

[18] Grant DF, Hammock BD (1992). Genetic and molecular evidence for a trans-acting regulatory locus controlling glutathione S-transferase-2 expression in *Aedes aegypti*. Mol Gen Genet.

[19] Ranson H, Rossiter L, Ortelli F, Jensen B, Wang X, Roth CW (2001). Identification of a novel class of insect glutathione S-transferases involved in resistance to DDT in the malaria vector *Anopheles gambiae*. Biochem J..

[20] Tikar SN, Kumar A, Prasad GB, Prakash S (2009). Temephos-induced resistance in *Aedes aegypti* and its cross-resistance studies to certain insecticides from India. Parasitol Res.

[21] David JP, Strode C, Vontas J, Nikou D, Vaughan A, Pignatelli PM (2005). The *Anopheles gambiae* detoxification chip: a highly specific microarray to study metabolic-based insecticide resistance in malaria vectors. Proc Natl Acad Sci USA.

[22] De Marco L, Sassera D, Epis S, Mastrantonio V, Ferrari M, Ricci I (2017). The choreography of the chemical defensome response to insecticide stress: insights into the *Anopheles stephensi* transcriptome using RNA-Seq. Sci Rep..

[23] Lumjuan N, Rajatileka S, Changsom D, Wicheer J, Leelapat P, Prapanthadara LA (2011). The role of the *Aedes aegypti* Epsilon glutathione transferases in conferring resistance to DDT and pyrethroid insecticides. Insect Biochem Mol Biol.

[24] Weill M, Berthomieu A, Berticat C, Lutfalla G, Nègre V, Pasteur N (2004). Insecticide resistance: a silent base prediction. Curr Biol.

[25] Epis S, Porretta D, Mastrantonio V, Comandatore F, Sassera D, Rossi P (2014). ABC transporters are involved in defense against permethrin insecticide in the malaria vector *Anopheles stephensi*. Parasit Vectors..

[26] Epis S, Porretta D, Mastrantonio V, Urbanelli S, Sassera D, De Marco L (2014). Temporal dynamics of the ABC transporter response to insecticide treatment: insights from the malaria vector *Anopheles stephensi*. Sci Rep..

[27] Porretta D, Epis S, Mastrantonio V, Ferrari M, Bellini R, Favia G (2016). How heterogeneous is the involvement of ABC transporters against insecticides?. Acta Trop.

[28] Mastrantonio V, Ferrari M, Epis S, Negri A, Scucciamarra G, Montagna M (2017). Gene expression modulation of ABC transporter genes in response to permethrin in adults of the mosquito malaria vector *Anopheles stephensi*. Acta Trop.

[29] Mastrantonio V, Ferrari M, Negri A, Sturmo T, Favia G, Porretta D (2019). Insecticide exposure triggers a modulated expression of abc transporter genes in larvae of *Anopheles gambiae* s.s. Insects..

[30] Zhang X, Zhang J, Zhu KY (2010). Chitosan/double-stranded RNA nanoparticle-mediated RNA interference to silence chitin synthase genes through larval feeding in the African malaria mosquito (*Anopheles gambiae*). Insect Mol Biol.

[31] Singh AD, Wong S, Ryan CP, Whyard S (2013). Oral delivery of double-stranded RNA in larvae of the yellow fever mosquito, *Aedes aegypti*: implications for pest mosquito control. J Insect Sci..

[32] Ingham VA, Jones CM, Pignatelli P, Balabanidou V, Vontas J, Wagstaff SC (2014). Dissecting the organ specificity of insecticide resistance candidate genes in *Anopheles gambiae*: known and novel candidate genes. BMC Genomics..

[33] Kumar P, Pandit SS, Steppuhn A, Baldwin IT (2014). Natural history-driven, plant-mediated RNAi-based study reveals CYP6B46′s role in a nicotine-mediated antipredator herbivore defense. Proc Natl Acad Sci USA.

[34] Whyard S, Erdelyan CN, Partridge AL, Singh AD, Beebe NW, Capina R (2015). Silencing the buzz: a new approach to population suppression of mosquitoes by feeding larvae double-stranded RNAs. Parasit Vectors..

[35] Mysore K, Hapairai LK, Sun L, Harper EI, Chen Y, Eggleson KK (2017). Yeast interfering RNA larvicides targeting neural genes induce high rates of *Anopheles* larval mortality. Malar J..

[36] Fillinger U, Lindsay SW (2011). Larval source management for malaria control in Africa: myths and reality. Malar J..

[37] WHO. Larval source management: a supplementary malaria vector control measure: an operational manual. World Health Organization; 2013. http://www.who.int/iris/handle/10665/85379.

[38] Summerton J, Weller D (1997). Morpholino antisense oligomers: design, preparation, and properties. Antisense Nucleic Acid Drug Dev.

[39] Summerton J (2007). Morpholino, siRNA, and S-DNA compared: impact of structure and mechanism ofaction on off-target effects and sequence specificity. Curr Top Med Chem.

[40] Luo Y, Wang X, Wang X, Yu D, Chen B, Kang L (2013). Differential responses of migratory locusts tosystemic RNA interference via double-stranded RNA injection and feeding. Insect Mol Biol.

[41] Pietri JE, Cheung KW, Luckhart S (2014). Knockdown of mitogen-activated protein kinase (MAPK) signalling in the midgut of *Anopheles stephensi* mosquitoes using antisense morpholinos. Insect Mol Biol.

[42] Moulton JD (2016). Guide for morpholino users: toward therapeutics. J Drug Discov Dev Deliv..

[43] Layden MJ, Rottinger E, Wolenski FS, Gilmore TD, Martindale MQ (2013). Microinjection of mRNA or morpholinos for reverse genetic analysis in the starlet sea anemone, *Nematostella vectensis*. Nat Protoc..

[44] Melvin VS, Feng W, Hernandez-Lagunas L, Artinger KB, Williams T (2013). A Morpholino-based screen to identify novel genes involved in craniofacial morphogenesis. Dev Dyn.

[45] Arora V, Knapp DC, Reddy MT, Weller DD, Iversen PL (2002). Bioavailability and efficacy of antisense morpholino oligomers targeted to c-myc and cytochrome P-450 3A2 following oral administration in rats. J Pharm Sci.

[46] Weidinger G, Stebler J, Slanchev K, Dumstrei K, Wise C, Lovell-Badge R (2003). dead end, a novel vertebrate germ plasm component, is required for zebrafish primordial germ cell migration and survival. Curr Biol.

[47] Slanchev K, Stebler J, de la Cueva-Méndez G, Raz E (2005). Development without germ cells: the role of the germ line in zebrafish sex differentiation. Proc Natl Acad Sci USA.

[48] WHO (2005). Guidelines for laboratory and field testing of mosquito larvicides Document WHO/CDS/WHOPES/GCDPP/13.

[49] Capone A, Ricci I, Damiani C, Mosca M, Rossi P, Scuppa P (2013). Interactions between *Asaia*, *Plasmodium* and *Anopheles*: new insights into mosquito symbiosis and implications in malaria symbiotic control. Parasit Vectors..

[50] Yamamoto DS, Sumitani M, Kasashima K, Sezutsu H, Matsuoka H (2016). Inhibition of malaria infection in transgenic anopheline mosquitoes lacking salivary gland cells. PLoS Pathog.

[51] Morcos PA, Li Y, Jiang S (2008). Vivo-Morpholinos: a non-peptide transporter delivers Morpholinos into a wide array of mouse tissues. Biotechniques.

[52] Li T, Liu L, Zhang L, Liu N (2014). Role of G-protein-coupled receptor-related genes in insecticide resistance of the mosquito, *Culex quinquefasciatus*. Sci Rep..

[53] Li T, Cao C, Yang T, Zhang L, He L, Xi Z (2015). A G-protein-coupled receptor regulation pathway in cytochrome P450-mediated permethrin-resistance in mosquitoes, *Culex quinquefasciatus*. Sci Rep..

[54] Guo Z, Kang S, Zhu X, Xia J, Wu Q, Wang S (2015). The novel ABC transporter ABCH1 is a potential target for RNAi-based insect pest control and resistance management. Sci Rep..

[55] Huvenne H, Smagghe G (2010). Mechanisms of dsRNA uptake in insects and potential of RNAi for pest control: a review. J Insect Physiol.

[56] Whangbo JS, Hunter CP (2008). Environmental RNA interference. Trends Genet.

[57] Feinberg EH, Hunter CP (2003). Transport of dsRNA into cells by the transmembrane protein SID-1. Science.

[58] Winston WM, Sutherlin M, Wright AJ, Feinberg EH, Hunter CP (2007). *Caenorhabditis elegans* SID-2 is required for environmental RNA interference. Proc Natl Acad Sci USA.

[59] Tomoyasu Y, Miller SC, Tomita S, Schoppmeier M, Grossmann D, Bucher G (2008). Exploring systemic RNA interference in insects: a genome-wide survey for RNAi genes in *Tribolium*. Genome Biol.

[60] Pillai AB, Nagarajan U, Mitra A, Krishnan U, Rajendran S, Hoti SL (2017). RNA interference in mosquito: understanding immune responses, double-stranded RNA delivery systems and potential applications in vector control. Insect Mol Biol.

[61] Phanse Y, Dunphy BM, Perry JL, Airs PM, Paquette CC, Carlson JO (2015). Biodistribution and toxicity studies of PRINT hydrogel nanoparticles in mosquito larvae and cells. PLoS Negl Trop Dis..

[62] Araujo RN, Santos A, Pinto FS, Gontijo NF, Lehane MJ, Pereira MH (2006). RNA interference of the salivary gland nitrophorin 2 in the triatomine bug *Rhodnius prolixus* (Hemiptera: Reduviidae) by dsRNA ingestion or injection. Insect Biochem Mol Biol.

[63] Joga MR, Zotti MJ, Smagghe G, Christiaens O (2016). RNAi efficiency, systemic properties, and novel delivery methods for pest insect control: what we know so far. Front Physiol..

[64] Airs PM, Bartholomay LC (2017). RNA Interference for mosquito and mosquito-borne disease control. Insects..

[65] Fischer JR, Zapata F, Dubelman S, Mueller GM, Uffman JP, Jiang C (2017). Aquatic fate of a double-stranded RNA in a sediment-water system following an over-water application. Environ Toxicol Chem.

[66] Cancino-Rodezno A, Alexander C, Villaseñor R, Pacheco S, Porta H, Pauchet Y (2010). The mitogen-activated protein kinase p38 pathway is involved in insect defense against Cry toxins from *Bacillus thuringiensis*. Insect Biochem Mol Biol.

[67] Rodríguez-Almazán C, Reyes EZ, Zúñiga-Navarrete F, Muñoz-Garay C, Gómez I, Evans AM (2012). Cadherin binding is not a limiting step for *Bacillus thuringiensis* subsp. *israelensis* Cry4Ba toxicity to *Aedes aegypti* larvae. Biochem J..

[68] Dass CR, Choong PF (2008). Chitosan-mediated orally delivered nucleic acids: a gutful of gene therapy. J Drug Target.

[69] Zhang X, Mysore K, Flannery E, Michel K, Severson DW, Zhu KY, Duman-Scheel M (2015). Chitosan/interfering RNA nanoparticle mediated gene silencing in disease vector mosquito larvae. J Vis Exp..

[70] Jeon SJ, Oh M, Yeo WS, Galvão KN, Jeong KC (2014). Underlying mechanism of antimicrobial activity of chitosan microparticles and implications for the treatment of infectious diseases. PLoS ONE.

[71] Stewart ZP, Oxborough RM, Tungu PK, Kirby MJ, Rowland MW, Irish SR (2013). Indoor application of attractive toxic sugar bait (ATSB) in combination with mosquito nets for control of pyrethroid-resistant mosquitoes. PLoS ONE.

[72] Van Ekert E, Powell CA, Shatters RG, Borovsky D (2014). Control of larval and egg development in *Aedes aegypti* with RNA interference against juvenile hormone acid methyl transferase. J Insect Physiol.

[73] Zhang Y, Zhang G, Li Y, Hu Y (2013). Probing the binding of insecticide permethrin to calf thymus DNA by spectroscopic techniques merging with chemometrics method. J Agric Food Chem.

[74] Kasai S, Weerasinghe IS, Shono T (1998). P450 Monooxygenases are an important mechanism of permethrin resistance in *Culex quinquefasciatus* say larvae. Arch Insect Biochem Physiol.

[75] Hemingway J, Ranson H (2000). Insecticide resistance in insect vectors of human disease. Ann Rev Entomol..

[76] Kasai S, Shono T, Komagata O, Tsuda Y, Kobayashi M, Motoki M (2007). Insecticide resistance in potential vector mosquitoes for West Nile virus in Japan. J Med Entomol.

[77] Liu N (2015). Insecticide resistance in mosquitoes: impact, mechanisms, and research directions. Annu Rev Entomol.

